# E-M, an Engineered Endostatin with High ATPase Activity, Inhibits the Recruitment and Alternative Activation of Macrophages in Non-small Cell Lung Cancer

**DOI:** 10.3389/fphar.2017.00532

**Published:** 2017-08-09

**Authors:** Min Xu, Shaosen Zhang, Lin Jia, Shan Wang, Jie Liu, Xuhui Ma, Chunying Wang, Yan Fu, Yongzhang Luo

**Affiliations:** ^1^The National Engineering Laboratory for Anti-Tumor Protein Therapeutics, Tsinghua University Beijing, China; ^2^Beijing Key Laboratory for Protein Therapeutics, Tsinghua University Beijing, China; ^3^Cancer Biology Laboratory, School of Life Sciences, Tsinghua University Beijing, China

**Keywords:** endostatin, ATPase activity, tumor-associated macrophage, cell recruitment, alternative activation

## Abstract

Endostatin recently was reported by our laboratory to possess ATPase activity that is indispensable for its anti-angiogenesis and anti-tumor effects. An engineered endostatin, E-M, which owns higher ATPase activity exhibits stronger inhibitory effects on angiogenesis. Tumor-associated macrophages (TAMs), especially M2-polarized TAMs, contribute to tumor progression by promoting tumor cell proliferation, metastasis, angiogenesis, and immunosuppression, thus emerging as crucial targets for therapeutic intervention. Endostatin reportedly modulated functions of TAMs, but the detailed mechanisms remain unclear. Here, in our study, we demonstrated that E-M exhibited stronger inhibitory effects on macrophages than endostatin and other low ATPase mutants, which indicates that the ATPase activity is required for the inhibitory effects of endostatin on TAMs. Moreover, we elucidated that endostatin co-receptor, nucleolin and integrin α5β1, overexpressed on the surface of M2 macrophages, facilitated the internalization of E-M via the caveolae/lipid raft- and clathrin-dependent pathways. E-M inhibited the migration of TAMs through blockade of p38 MAP kinase and Erk1/2 signaling pathways, and prevented the alternative activation of TAMs. As a result, TAM-induced tumor cell proliferation and angiogenic activities *in vitro* were dramatically suppressed by E-M. In a transplanted non-small cell lung cancer model, E-M remarkably decreased the density of intratumoral macrophages and blood vessels, leading to tumor regression. This study unravels a novel mechanism of endostatin on regulating TAM recruitment and polarization, and suggests that E-M is a remarkably promising and multifunctional anti-tumor agent.

## Introduction

Macrophages are important tissue-resident immune cells which participate in maintaining homeostasis and defensing against pathogens (Murray and Wynn, 2011). Plasticity and diversity of polarization is a hallmark of macrophages. Classically activated macrophages and alternatively activated macrophages are normally referred to as M1 macrophages and M2 macrophages, respectively. M1 macrophages participating in Th1 response are involved in anti-tumor immunity and pro-inflammatory function, whereas M2 macrophages exhibit pro-tumor and anti-inflammatory effects (Mantovani et al., 2004; Sica and Bronte, 2007; Ruffell and Coussens, 2015). During tumor progression, large amounts of macrophages infiltrate malignant tumors and these cells are commonly termed tumor-associated macrophages (TAMs) (Lewis and Pollard, 2006). Many studies have shown that high densities of M2 TAMs are closely related to poor clinical prognosis (Chen et al., 2003; Ruffell and Coussens, 2015; Mei et al., 2016).

Tumor-associated macrophages often display similar characteristics typical of M2 macrophages. These cells can enhance tumor angiogenesis, immunosuppression, tumor cell invasion and metastasis via the production of different cytokines and chemokines such as VEGF-A, CCL17/CCL22, and EGF (Columba-Cabezas et al., 2002; Goswami et al., 2005; Murdoch et al., 2008). It is well-known that colony-stimulating factor 1 (CSF-1) and CSF-1R modulate the motility, differentiation and survival of macrophages (Hume and MacDonald, 2012). Monoclonal antibodies or selective inhibitors of CSF-1R have been developed by pharmaceutical companies. RG7155, a humanized monoclonal antibody of CSF-1R, strikingly reduces the CSF-1R^+^CD163^+^ macrophage infiltration in patients with diffuse-type giant cell tumor and increases CD8^+^ T cells and NK cells in tumor tissues (Ries et al., 2014). The tyrosine kinase inhibitor of CSF-1R, PLX3397, reportedly improves the efficacy of immunotherapy by decreasing immunosuppressive TAMs in tumor tissues (Mok et al., 2014). Moreover, activation of caspase-8-dependent apoptotic pathway in TAMs by the compound Trabectedin is beneficial for the treatment of soft tissue sarcoma and platinum-sensitive ovarian cancer patients (Germano et al., 2013). Therefore, inhibitions of recruitment, alternative activation and survival of TAMs are considered as promising approaches for cancer therapy.

Endostatin, a 20 kD protein cleaved from C-terminal domain of collagen XVIII, is an endogenous anti-angiogenic protein (O’Reilly et al., 1997). Our group previously demonstrated that correct refolding and N-terminal integrity are extremely important for the biological functions of endostatin (Fu et al., 2009; Fu and Luo, 2010). Endostatin has been approved for the treatment of non-small cell lung cancer (NSCLC) patients by China Food and Drug Administration (CFDA) (Rong et al., 2012), and now becomes one of the most popular anti-angiogenesis cancer drugs in the Chinese market. It displays inhibitory effects on endothelial cells and initiates endothelial cell apoptosis by modulating the phosphorylation of voltage-dependent anion channel 1 (O’Reilly et al., 1997; Yamaguchi et al., 1999; Joki et al., 2001; Yuan et al., 2008). Internalization of endostatin is important for the physiological functions of endostatin (Dixelius et al., 2000; MacDonald et al., 2001). Before internalization, endostatin binds to its receptors such as integrin α5β1, MMP-2, and glypicans (Kim et al., 2000; Karumanchi et al., 2001; Rehn et al., 2001). Our group also discovered that endothelial cell surface nucleolin is a novel receptor of endostatin which mediates the anti-angiogenesis and anti-tumor activities of endostatin (Shi et al., 2007). Subsequent studies from our laboratory showed that specific internalization of endostatin into endothelial cells is orchestrated by integrin α5β1-nucleolin-urokinase plasminogen activator receptor (uPAR) receptor complex via caveolae/lipid raft- and clathrin-dependent pathways (Chen et al., 2011; Song et al., 2012). Moreover, increased uptake of endostatin by cholesterol-chelating agents such as nystatin or adding a macromolecule transduction domain (MTD) remarkably improves the therapeutic efficacy on tumor models (Chen et al., 2011; Lim et al., 2013). Recently, our group unexpectedly discovered that endostatin contains ATPase activity. Substituting the Walker A motif in endostatin with that of myosin which has high ATPase activity, we named it as E-M for short, endows it with a much higher ATPase activity. Compared to the wild type (WT) endostatin, the characteristic of higher ATPase activity makes E-M exhibit stronger inhibitory effects on both angiogenesis and tumor growth (Wang S. et al., 2015).

In addition to its inhibitory effects on endothelial cells, endostatin also exhibits effects on other cell types. Our group previously discovered that endostatin displays combined inhibitory functions on angiogenesis and adipogenesis, which protects mice from dietary-induced obesity and some related metabolic disorders such as glucose intolerance, insulin resistance and hepatic steatosis (Wang H. et al., 2015). Chen et al. (2016) found that endostatin can also suppress osteoclast formation. Moreover, endostatin also reportedly increased the infiltration of NK cells and CD8^+^ T cells in tumor tissues (Coutinho et al., 2007; Rocha et al., 2010). Gene therapy of endostatin regulated the alternative activation of macrophages but not macrophage recruitment (Foguer et al., 2016; Guo et al., 2016). Since TAMs are crucial targets for cancer therapeutics and E-M has a better bioactivity than WT endostatin, we speculate that E-M will display stronger inhibitory effects on TAMs and tumor angiogenesis, hence better tumor inhibition.

Here we report that the recombinant endostatin derivative E-M, which has higher ATPase activity, displays stronger inhibitory effects on both TAM’s recruitment and polarization, thereby leading to the suppression of tumor angiogenesis and tumor growth. These novel functions sheds new insights into the biological relevance of E-M, suggesting that E-M is a promising multifunctional anti-tumor reagent.

## Materials and Methods

### Cells and Reagents

A549, Raw 264.7, B16-F10, L929, SVEC4-10, and MS1 murine endothelial cells were purchased from the American Type Culture Collection (ATCC). A549 cells were cultured in RPMI-1640 (Wisent, St-Bruno, QC, Canada) supplemented with 10% FBS. RAW 264.7, B16-F10, L929, SVEC4-10, and MS1 cells were maintained in DMEM supplemented with 10% FBS. All cells were cultured under the condition of 37°C and 5% CO_2_ in the cell incubator (Thermo Fisher Scientific). The recombinant murine M-CSF and IL-4 were purchased from Sino Biological Inc (Beijing, China). The recombinant murine IL-13 was purchased from PeproTech (Rocky Hill, NJ, United States). Antibodies such as p38, p-p38, Erk1/2, p-Erk1/2 and Hsp90 were from Santa Cruz Biotechnology (Santa Cruz, CA, United States). Akt, p-Akt, 4E-BP1, p-4E-BP1, and Arginase-1 antibodies were from Cell Signaling Technology (Beverley, MA, United States). Integrin α5 and nucleolin antibodies were from Abcam (Cambridge, United Kingdom). Rabbit polyclone antibody for endostatin was from Oncogene Research Products (Cambridge, MA, United States). CD31 and F4/80 antibodies were from BD Bioscience (San Jose, CA, United States). CD11b-FITC and CD11b-PE-Cy7 antibodies for flow cytometry were from eBioscience (San Diego, CA, United States). F4/80-FITC and CD206-APC antibodies were from Biolegend (San Diego, CA, United States). HIF-1α antibody was from GeneTex (Irvine, TX, United States). FITC-linked anti-rat, FITC-linked anti-rabbit, TRITC-linked anti-mouse and Dylight 649-linked anti-rabbit IgG antibodies were from Beijing Cowin Biotech (Beijing, China).

### Immunofluorescence

Cells were fixed with 4% paraformaldehyde and washed with ice-cold PBS twice. For tumor tissues, slides of cryo-section were fixed in ice-cold acetone and washed in PBS. Then these samples were blocked with 10% goat serum and stained with primary antibodies. At last, fluorescein-conjugated secondary antibodies were added and fluorescent images were captured by Nikon A1 laser scanning confocal microscope. These images were then analyzed with Nikon image software NIS-Elements AR 3.0.

### Cell Apoptosis Assay

Bone marrow-derived macrophages (BMDMs) were treated with 20 and 40 μg/mL E-M for 24 h. Then these cells were harvested and washed in cold PBS. After centrifugation, 100 μL binding buffer was added to these cells. Then 5 μL Annexin V-FITC and 5 μL PI staining solution (Biotool, China) were added to the cell suspension. These cells were incubated for 15 min at room temperature. At last, 400 μL binding buffer was added to the cell suspension and cell apoptosis was analyzed by BD Calibur (BD Biosciences, San Jose, CA, United States).

### RNA Extraction and Analysis

Total RNA from cells was isolated with TRIzol (Life Technologies, Carlsbad, CA, United States) and subjected to cDNA synthesis with the First Strand cDNA Synthesis Kit (Fermentas, Hanover, MD, United States). Quantitative RT-PCR (qRT-PCR) was used to analyze mRNA expression levels. It was performed with 2×TransStart Green qPCR SuperMix (TransGen Biotech, Beijing, China). The reaction was run on the Mx3000P system (Stratagene, La Jolla, CA, United States). GAPDH or 18S was used as the control. Relative quantification was analyzed with the ΔΔCt method. The primers for qRT-PCR were listed in Supplementary Table 1.

### Western Blot

Western blot was performed following the previous report (Ding et al., 2010). In brief, cells were harvested, boiled, and subjected to 10% or 12% SDS-PAGE. Proteins in the gel were transferred to the PVDF membrane (Millipore, Bedford, MA, United States) and the membrane was blocked in 5% fat-reduced milk and incubated with primary antibodies overnight at 4°C. Then the membrane was incubated with HRP-conjugated goat anti-rabbit or goat anti-mouse secondary antibodies for 1 h at room temperature. At last, proteins on the membrane were detected with enhanced chemiluminescence system (Pierce Biotechnology, Radford, IL, United States) following the manufacturer’s protocol.

### MTT Assay

4 × 10^3^ cells were seeded in each well of 96-well plate. Cells were treated with E-M for 72 h. After the treatment, 10 μL MTT solution (Beyotime Biotechnology, China) in 100 μL DMEM was added to each well and the whole plate was incubated at 37°C for 4 h. Then 100 μL formazan solution was added to each well and incubated for another 4 h. At last, the absorbance of each well at 570 nm (OD_570_) was measured under Varioskan Flash (Thermo Fisher Scientific).

### Flow Cytometric Analysis

1 × 10^6^ cells in 100 μL PBS containing 3% FBS were stained with primary antibodies on ice for 30 min and washed with ice-cold PBS once. Then these cells were stained with fluorescein-conjugated secondary antibodies for 30 min and washed with ice-cold PBS twice. For fluorescein-labeled primary antibodies, cells were just stained with these antibodies and washed with PBS once. At last, 500 μL PBS was added to these cells and cells were analyzed with FACSAria III (BD Biosciences).

### Cell Migration Assay

Raw 264.7 cells or BMDMs were pre-treated with endostatin or different ATPase mutants for 1 h and 1 × 10^5^ cells were seeded in the upper chamber of 5 μm Millicell (Millipore). Recombinant proteins including WT endostatin, E-M, E176A, K96A, and K96R were added to the medium containing concentrated A549 conditioned medium (CM) or 2% FBS in the lower chamber. Cells were allowed to migrate for 6 h at 37°C. Then cells were fixed with 4% paraformaldehyde and stained with crystal violet (Sigma–Aldrich, St. Louis, MO, United States). Migrated cells were counted randomly in five independent fields under the Olympus IX71 optical microscope.

### Wound Healing Assay

SVEC4-10 cells were plated in the 6-well plate and allowed to grow to confluence. Then 10 μL pipette tips were used to scratch lines. BMDM CM from different treatment groups was added to these wells and cells were incubated at 37°C for 6 h. Images were taken with optical microscope and five independent were analyzed with Image-Pro Plus 6.0 software (Media Cybernetics, Silver Spring, MD, United States).

### Tubule Formation Assay

The tubule formation assay was performed as previously described (Huang et al., 2006). In brief, 100 μL Matrigel (Corning, NY, United States) was added to wells of 48-well plate and the plate was incubated at 37°C for 30 min. Then 3 × 10^4^ MS1 cells in the medium containing 2% FBS were added to each well and cultured at 37°C. After 3.5 h, tubule formation was observed and five independent fields were captured under the optical microscope. The tubule length was analyzed with Image-Pro Plus 6.0 software.

### BMDM Induction

Cells in the tibia and femur of C57BL/6 mice were flushed out with 10 mL DMEM. After red blood cells lysis, myeloid cells were incubated in medium containing 10% FBS and 100 ng/mL M-CSF or L929 CM. At the 4th day, fresh medium was added to these cells and incubated for another 3 days.

### Animal Studies

All animal experiments were approved by the Institutional Animal Care and Use Committees of Tsinghua University (Approval No.14-LYZ4). 1 × 10^6^ A549 cells were inoculated subcutaneously into the right axilla of female BALB/c nude mice (Vital River, China). When these tumors grew to 100 mm^3^, mice were separated randomly into two groups (*n* = 5 mice/group). For the E-M treatment group, 12 mg/kg E-M was administered to mice intravenously every other day and the treatment was lasted for 12 days. For liposome treatment group, 200 μL PBS or clodronate liposomes were injected to mice (*n* = 5 mice/group) 6 days before the tumor implantation. The injection was given every 3 days and tumors were allowed to grow for 12 days. Tumor growth was monitored and tumor volumes were calculated by the formula: volume = 0.52ab^2^ (*a* represents the long diameter and *b* indicates the short diameter).

### Clodronate Encapsulation

PBS and clodronate liposomes were prepared following the previous report (Van Rooijen and Sanders, 1994). Under the protection of argon, clodronate was encapsulated in liposomes that consisted of phosphatidylcholine (Lipoid, Germany) and cholesterol (Sigma–Aldrich).

### Statistical Analysis

All experimental data were presented as mean ± SD or SEM. A two-tailed Student’s *t*-test was used to assess the statistical differences for two-group comparisons. For comparing three or more groups, an one-way analysis of variance (ANOVA) was applied to assess the statistical differences. *P* < 0.05 was regarded to be significant.

## Results

### E-M Exhibits Strong Inhibitory Effects on Macrophages and Interacts with Both Nucleolin and Integrin α5β1

**FIGURE 1:**
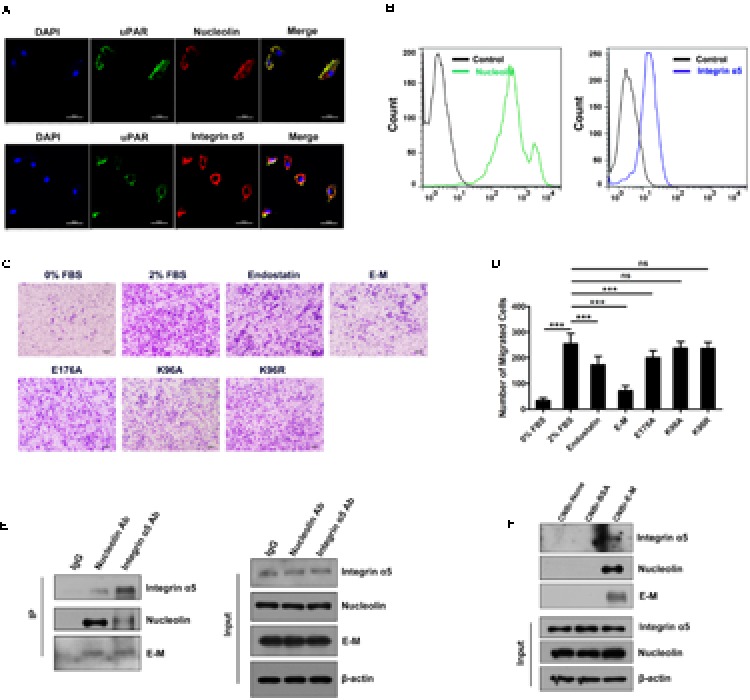
E-M exhibits strong inhibitory effects on macrophages and interacts with both nucleolin and integrin α5β1. **(A)** Immunofluorescent images showing that uPAR, nucleolin and integrin α5 were co-localized on BMDM surface. **(B)** Flow cytometric analysis showing the presence of nucleolin and integrin α5 on BMDM surface. **(C)** Representative images of effects of endostatin, E-M and low ATPase mutants (E176A, K96A, and K96R) on BMDM migration determined by modified Boyden chamber assay; Scale bar = 100 μm. BMDMs were pre-treated with 40 μg/mL different recombinant proteins for 1 h. Then 2% FBS was added to the lower chamber to induce BMDM migration. **(D)** Quantified result of **(C)**. **(E)** Immunoprecipitation showing both nucleolin and integrin α5 were able to interact with E-M. **(F)** Pull-down assay showing the interaction between E-M and nucleolin or integrin α5. E-M and BSA were linked to CNBr-activated sepharose, respectively. BMDM cell lysates were incubated with these sepharose. Then samples were immunoblotted with antibodies against nucleolin and integrin α5. Data were representative of mean ± SD from at least three independent experiments. *P*-value: One-way ANOVA; ^∗∗∗^*P* < 0.001; ns, not significant.

As the integrin α5β1-nucleolin-uPAR co-receptor complex was identified to mediate endostatin internalization and nuclear translocation of endostatin in endothelial cells (Rehn et al., 2001; Shi et al., 2007; Song et al., 2012), we examined the expression of nucleolin, integrin α5 and uPAR in BMDMs and Raw 264.7 cells, and found that all these proteins were expressed on the cell surface (**Figure 1A** and Supplementary Figure 1A). Flow cytometric analysis confirmed these results (**Figure 1B** and Supplementary Figure 1B). We also isolated TAMs from A549 tumor tissues and found that both nucleolin and integrin α5 were expressed on the surface of TAMs (Supplementary Figure 1C). To test whether ATPase activity is crucial for the bioactivities of endostatin on macrophages, we treated BMDMs and Raw 264.7 cells with WT endostatin, E-M and other mutants with lower ATPase activity (K176A, K96A, and K96R) in *in vitro* migration assay. Compared to endostatin, E-M exhibited a much stronger inhibitory effect on macrophage migration, whereas these low-ATPase mutants had no or only minor effects on macrophage migration (**Figures 1C,D** and Supplementary Figures 1D,E). Therefore for the rest of this study we mainly focused on the recombinant endostatin derivative E-M. As no pronounced changes of the tertiary structure happened in E-M (Wang S. et al., 2015), we assumed that nucleolin and integrin α5β1 could interact with E-M and they were also E-M receptors. The immunoprecipitation result showed that nucleolin and integrin α5 both directly interacted with E-M in BMDMs (**Figure 1E**). We also linked E-M to CNBr-activated Sepharose 4B column and incubated it with BMDM lysates. After elution, nucleolin and integrin α5 were detected in the eluted solution which further confirmed the interaction between E-M and nucleolin or integrin α5 (**Figure 1F**). Taken together, the ATPase activity is necessary for the biofunctions of endostatin on macrophage functions, and nucleolin and integrin α5β1 expressed on macrophage surface were also E-M receptors.

### E-M Can Be Internalized into Macrophages via Cell Surface Nucleolin and Integrin α5β1 in Caveolae/Lipid Raft- and Clathrin-Dependent Pathways

**FIGURE 2:**
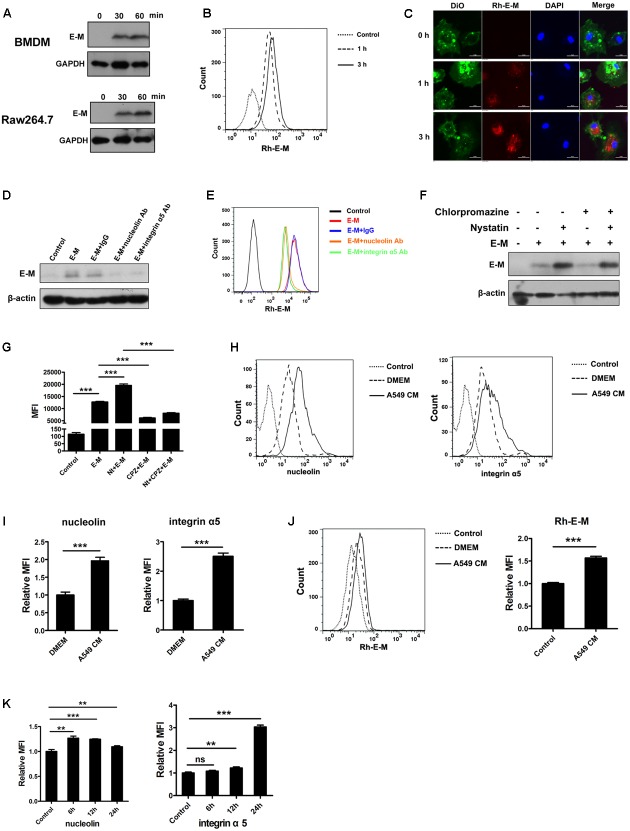
E-M can be internalized into macrophages via cell surface nucleolin and integrin α5β1 in caveolae/lipid raft- and clathrin-dependent pathways. **(A)** Western blot indicating the internalization of E-M into BMDMs and Raw 264.7 cells in a time-dependent manner. The culture medium was replaced with fresh DMEM without FBS. Then 5 μg/mL E-M was added to the medium. After 30 or 60 min, cells were washed with ice cold acid buffer (pH = 3.5) twice and PBS once. Western blot was used to detect the internalization of E-M. **(B)** Flow cytometric analysis showing the internalization of E-M into BMDMs. **(C)** Representative images of immunofluorescence showing the localization of Rh-E-M in BMDMs. Blue: DAPI, green: DiO, red: Rh-E-M; Scale bar = 50 μm. **(D)** Blocking nucleolin and integrin α5 with respective antibodies and detecting the internalization of E-M into BMDMs. **(E)** Flow cytometric analysis showing the internalization of E-M in BMDMs after blockade of nucleolin and integrin α5 with respective antibodies. **(F)** Western blot indicating effects of caveolin inhibitor nystatin (50 μg/mL) and clathrin inhibitor chlorpromazine (6 μg/mL) on E-M internalization. **(G)** Flow cytometric analysis showing the effects of nystatin and chlorpromazine on E-M internalization. **(H)** Flow cytometric analysis showing expression levels of nucleolin and integrin α5 on BMDM surface after the treatment with A549 CM. **(I)** Quantified result of MFI in **(H)**. **(J)** Flow cytometric result displaying the internalization of Rh-E-M into BMDMs after the treatment of A549 CM. **(K)** Flow cytometric analysis showing expression levels of nucleolin and integrin α5 on BMDM surface after the treatment with IL-4 (20 ng/mL) and IL-13 (20 ng/mL) for 6, 12, and 24 h. Data were representative of mean ± SD from at least three independent experiments. *P*-value: Student’s *t*-test for two groups and One-way ANOVA for more than two groups; ^∗∗^*P* < 0.01, ^∗∗∗^*P* < 0.001; ns, not significant.

Previous studies showed that internalization of endostatin via its receptors was essential for its biological functions in endothelial cells and adipocytes (Dixelius et al., 2000; Wang H. et al., 2015). We hypothesized that E-M could also be internalized into macrophages. After treating BMDMs and Raw 264.7 cells with E-M, we found that E-M was uptaken by these cells (**Figure 2A**). Flow cytometric results showed that rhodamine-conjugated E-M (Rh-E-M) was internalized into BMDMs and Raw264.7 cells in a time-dependent manner (**Figure 2B** and Supplementary Figure 2A). After acid buffer washing, almost no E-M existed on the cell membrane (**Figure 2C** and Supplementary Figures 2B–D). After blockade of nucleolin and integrin α5 with antibodies, the internalization of E-M was significantly inhibited in BMDMs (**Figures 2D,E**). Our group previously reported that blockade of caveolae/lipid raft pathway accelerated endostatin internalization through the more efficient clathrin pathway (Chen et al., 2011). Similar results were obtained in BMDMs. The internalization of E-M was switched to clathrin pathway after nystatin treatment and the internalization was enhanced. However, blockade of clathrin pathway with chlorpromazine (CPZ) reversed this effect (**Figures 2F,G** and Supplementary Figure 2E). TAMs express genes typical of M2 macrophages and closely resemble M2 type macrophages. Our findings observed that after the treatment with A549 CM, the amount of nucleolin and integrin α5 on the surface of M2 type BMDMs or Raw 264.7 cells was increased (**Figures 2H,I** and Supplementary Figure 2F). The internalization of E-M was also enhanced (**Figure 2J** and Supplementary Figures 2G,H). Moreover, we induced M2 macrophages with IL-4 and IL-13 and similar results were obtained in these M2 macrophages (**Figure 2K** and Supplementary Figure 2I). In conclusion, E-M is internalized into macrophages via nucleolin and integrin α5β1 in caveolin- and clathrin-dependent pathways, and up-regulation of these two receptors on the surface of M2 macrophages enhances the internalization of E-M.

### E-M Exhibits Its Inhibitory Effects on TAM Motility Both *In Vitro* and *In Vivo*

**FIGURE 3:**
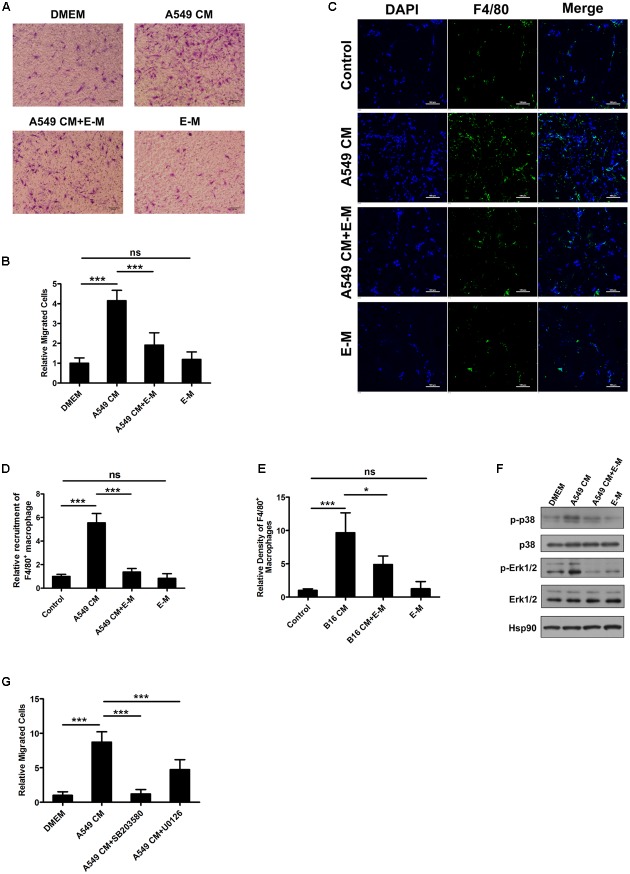
E-M exhibits its inhibitory effects on TAM motility both *in vitro* and *in vivo*. **(A)** Representative images of effects of E-M on BMDM migration determined by modified Boyden chamber assay; Scale bar = 100 μm. BMDMs were pre-treated with 40 μg/mL recombinant E-M for 1 h. Then concentrated A549 CM with or without E-M was added to the lower chamber to induce BMDM migration. **(B)** Quantified result of **(A)**. **(C)** Representative images of the density of F4/80^+^ macrophages recruited by A549 CM in Matrigel plugs; Scale bar = 100 μm. **(D)** Quantitation of recruited F4/80^+^ macrophages in **(C)**. **(E)** Quantified result of recruited macrophages by B16-F10 CM. **(F)** Western blot showing the effect of E-M on the activation of p38 and Erk1/2 induced by A549 CM. BMDMs were starved overnight in DMEM without FBS. Before stimulating BMDMs with A549 CM for 10 min, BMDMs were pre-treated with 20 and 40 μg/mL E-M for 1 h. Then cell lysates were immunoblotted for detecting the activation of p38 and Erk1/2. **(G)** The effects of p38 inhibitor SB203580 (10 μM) and Erk1/2 inhibitor U0126 (10 μM) on BMDM migration induced by A549 CM. BMDMs were pre-treated with SB203580 and U0126 for 30 min. Then concentrated A549 CM with or without inhibitors was added to the lower chamber to induce BMDM migration. Date were representative of mean ± SD from at least three independent experiments. *P*-value: One-way ANOVA; ^∗^*P* < 0.05, ^∗∗∗^*P* < 0.001; ns, not significant.

In malignant tumor tissues, the recruitment of TAMs is driven by chemotactic factors mainly derived from tumor cells. Therefore we induced macrophage motility with A549 CM in the cell migration assay and found that enhanced cell migration was significantly suppressed by E-M (**Figures 3A,B**). In the Matrigel plug assay, A549 CM and B16-F10 CM recruited a large number of F4/80^+^ macrophages into plugs but E-M blocked the macrophage recruitment (**Figures 3C–E** and Supplementary Figure 3A). Moreover, the decreased recruitment was not due to cell apoptosis induced by E-M (Supplementary Figure 3B). Activation of Erk1/2 and p38 MAP kinase pathways reportedly mediates the chemotactic migration in macrophages (Chiou et al., 2004). We found that activation of p38 and Erk1/2 in BMDMs and Raw 264.7 cells were both inhibited after E-M treatment (**Figure 3F** and Supplementary Figure 3C). To further confirm that activation of p38 and Erk1/2 mediated the BMDM migration process induced by A549 CM, we treated BMDMs with inhibitors SB203580 and U0126 in the presence of A549 CM. Both inhibitors remarkably suppressed BMDM migration induced by A549 CM (**Figure 3G** and Supplementary Figure 3D). In summary, E-M blocks the recruitment of macrophages both *in vitro* and *in vivo* through inhibiting the activation of p38 MAP kinase and Erk1/2 signaling pathways.

### E-M Inhibits the Switch of Macrophage Polarization toward M2 Phenotype

**FIGURE 4:**
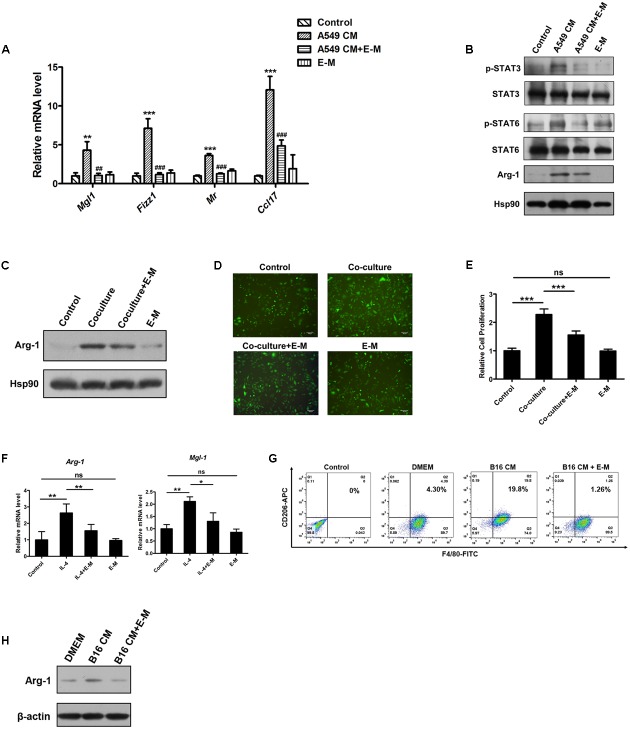
E-M inhibits the switch of macrophage polarization toward M2 phenotype. **(A)** qRT-PCR detecting genes of M2 markers in BMDMs after the treatment of A549 CM and E-M. **(B)** Western blot showing the activation of STAT3, STAT6 and the expression of Arg-1 in BMDMs after the treatment of A549 CM either alone or in the presence of E-M. **(C)** Western blot displaying the effect of E-M on the expression of Arg-1 in BMDMs in A549 cell-BMDM co-culture system. **(D)** Representative fluorescent images showing the number of A549-GFP cells in the A549 cell-BMDM co-culture system; Scale bar = 100 μm. A549-GFP cells were co-cultured with BMDMs for 12 h either alone or in the presence of E-M. **(E)** Quantitative result of A549-GFP cell number in **(D)**. **(F)** BMDMs were pre-treated with E-M for 1 h. Then IL-4 (40 ng/mL) was added to induce M2 macrophages. qRT-PCR was used to detect genes of M2 markers. **(G)** Flow cytometric result showing the effects of B16-F10 CM either alone or in the presence of E-M on alternative activation of BMDMs. **(H)** Western blotting result showing the effects of B16-F10 CM either alone or in the presence of E-M on the expression of Arg-1 in BMDMs. Data were representative of mean ± SD from at least three independent experiments. *P*-value: One-way ANOVA; ^∗^*P* < 0.05, ^∗∗^*P* < 0.01, ^∗∗∗^*P* < 0.001, ^##^*P* < 0.01, ^###^*P* < 0.001; ns, not significant.

To induce M2-polarized macrophages, we treated BMDMs with A549 CM and observed that the expression of M2 markers, including *Mgl1*, *Fizz1*, *Mr*, *Ccl17*, and ARG-1, were up-regulated. However, pre-treatment with E-M prevented the up-regulation of these markers (**Figures 4A,B**). During the polarization switch process, STAT3 and STAT6 were selectively activated in macrophages (Sica and Bronte, 2007). However, E-M could block the activation of STAT3 and STAT6 in BMDMs (**Figure 4B**). Compared to WT endostatin, E-M was also found to have stronger inhibitory effects on M2 polarization (Supplementary Figure 4A). In BMDM-A549 (GFP^+^) cell co-culture system, Arg-1 was also up-regulated in BMDMs but E-M reversed this effect (**Figure 4C**). Moreover, BMDMs enhanced the proliferation of A549 cells after 12 h in the co-culture system but the proliferation rate was reduced in the presence of E-M (**Figures 4D,E**). To exclude the direct influence on A549 cells, MTT assay was used to examine the effects of E-M on A549 cells and we found that E-M had no effects on A549 cell proliferation (Supplementary Figure 4B). Our findings also discovered that IL-4-induced M2 polarization was partially blocked by E-M (**Figure 4F**). Moreover, B16-F10 CM induced alternative activation of BMDMs was suppressed by E-M (**Figures 4G,H**). In conclusion, E-M can indeed inhibit the alternative activation of macrophages and M2 macrophage-induced tumor cell proliferation.

### E-M Suppresses the Pro-angiogenic Effects of TAMs

**FIGURE 5:**
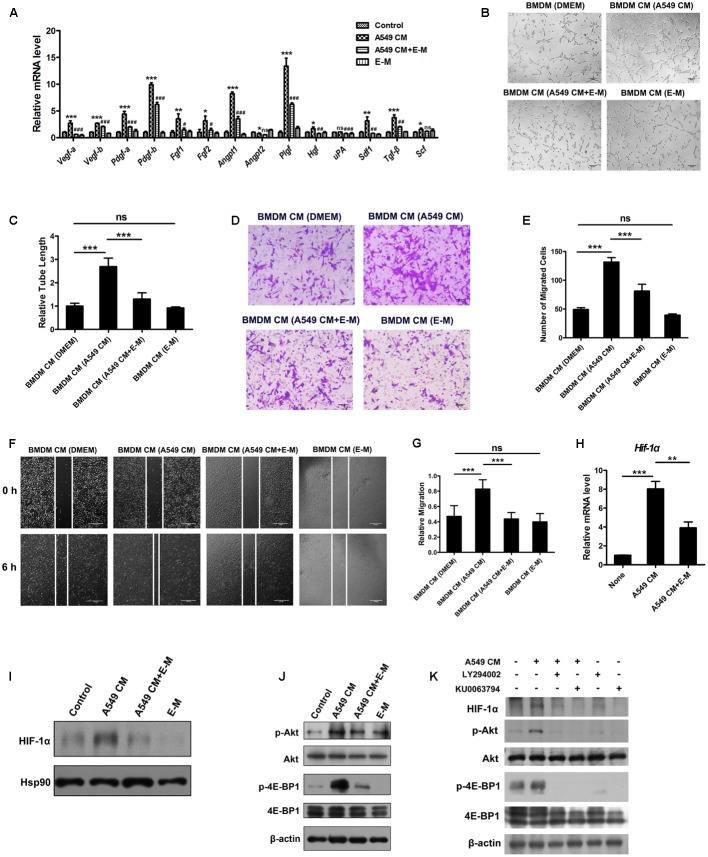
E-M suppresses the pro-angiogenic effects of TAMs. **(A)** qRT-PCR result showing the effect of E-M on the expression of pro-angiogenic cytokines in BMDMs which were treated with A549 CM. BMDMs were pre-treated with E-M for 1 h and then treated with A549 CM either alone or in the presence of E-M for 12 h. **(B)** Representative images showing BMDM CM from different groups on tubule formation. BMDMs were treated with A549 CM either alone or in the presence of E-M for 12 h. Then BMDMs were starved overnight and BMDM CM from each group was collected. These CM was used to treat MS1 cells for detection of tubule formation ability; Scale bar = 100 μm. **(C)** Quantified result of **(B)**. **(D)** Representative images showing the effect of BMDM CM from different groups on SVEC4-10 migration determined by Boyden chamber assay; Scale bar = 100 μm. **(E)** Quantified result of **(D)**. **(F)** Representative images displaying the effect of BMDM CM from different groups on SVEC4-10 motility determined by wound healing assay; Scale bar = 100 μm. **(G)** Quantified result of **(F)**. **(H)** qRT-PCR result indicating the effect of A549 CM either alone or in the presence of E-M on HIF-1α expression. **(I)** Western blot showing the effect of A549 CM either alone or in the presence of E-M on HIF-1α expression. **(J)** Western blot showing the effect of A549 CM either alone or in the presence of E-M on activation of Akt and 4E-BP1 in BMDMs. **(K)** Western blot showing the effect of A549 CM either alone or in the presence of PI3K inhibitor LY294002 (10 μM) or mTOR inhibitor KU0063794 (10 μM) on HIF-1α expression in BMDMs. Data were representative of mean ± SD from at least three independent experiments. *P*-value: One-way ANOVA; ^#^*P* < 0.05, ^##^*P* < 0.01, ^###^*P* < 0.001, ^∗^*P* < 0.05, ^∗∗^*P* < 0.01, ^∗∗∗^*P* < 0.001; ns, not significant.

Tumor-associated macrophages are well-known to promote tumor angiogenesis through production of different pro-angiogenic factors (Murdoch et al., 2008; Ruffell and Coussens, 2015). To detect whether E-M could inhibit pro-angiogenic effects of TAMs, we treated A549 CM-stimulated BMDMs with E-M. Several well-known pro-angiogenic factors such as VEGF-A, PDGF-B and placental growth factor (PlGF) were up-regulated after A549 CM treatment, whereas E-M blocked this effect (**Figure 5A**). After A549 CM treatment, we collected the BMDM CM in different treatment groups and treated endothelial cells with these CM. The tube formation ability of MS1 cells was enhanced in A549 CM pre-treatment group but this effect was abolished in the E-M pre-treatment group (**Figures 5B,C**). Moreover, consistent results were obtained in endothelial cell migration and wound healing assays. BMDM CM from A549 CM pre-treatment group enhanced the motility of endothelial cells but CM from E-M pre-treatment group had no such effect (**Figures 5D–G**). It was reported before that the transcription factor HIF-1α participated in regulation of pro-angiogenic factors production (Liu et al., 2008; Weidemann and Johnson, 2008). Our findings found that the expression of HIF-1α was up-regulated in mRNA and protein levels by A549 CM but it was suppressed by E-M (**Figures 5H,I**). PI3K/Akt and mTOR signaling pathways were known to regulate the HIF-1α expression in endothelial cells (Song et al., 2009). Then we detected whether these two pathways were also activated in BMDMs after A549 CM treatment. We found that the phosphorylation of Akt and 4E-BP1, the downstream target of mTOR, was increased after A549 CM treatment but the activation was blocked by E-M (**Figure 5J**). After the blockade of activation of PI3K/Akt and mTOR with their respective inhibitors LY294002 and KU0063794, the expression of HIF-1α induced by A549 CM in BMDMs was significantly down-regulated (**Figure 5K**). Taken together, E-M can abolish the pro-angiogenic effects of tumor cell-induced TAMs.

### E-M Inhibits the Recruitment of Macrophages and Tumor Angiogenesis *In Vivo*

**FIGURE 6:**
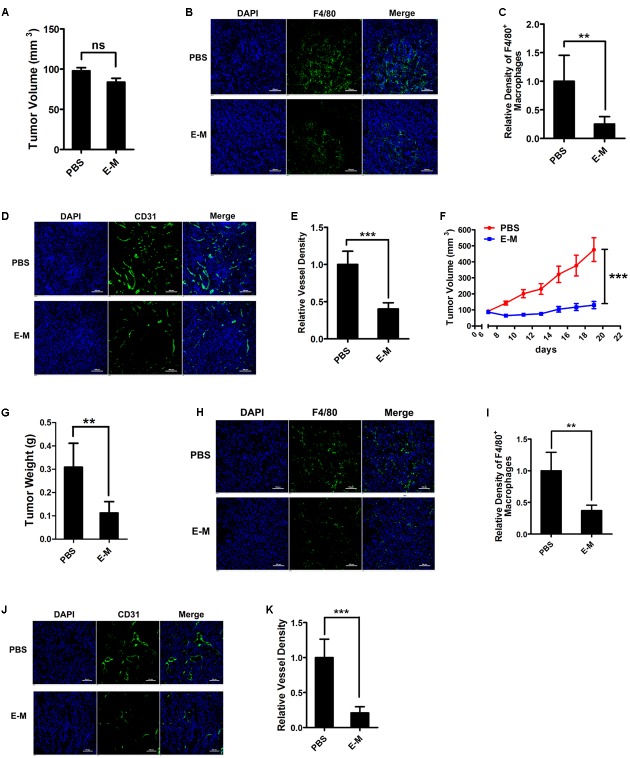
E-M inhibits the recruitment of macrophages and tumor angiogenesis *in vivo*. **(A)** Tumor volumes of A549 after treatment of PBS or E-M for 6 days (*n* = 5 mice/group). **(B)** Representative images of immunofluorescence showing the density of F4/80^+^ macrophages in A549 tumor tissues; Blue: DAPI, green: F4/80; Scare bar = 100 μm. **(C)** Quantitation of the density of macrophages in **(B)**. **(D)** Representative images of immunofluorescence displaying the density of blood vessels in A549 tumor tissues via detecting CD31^+^ blood vessels; Blue: DAPI, green: CD31; Scare bar = 100 μm. **(E)** Quantified result of **(D)**. **(F)** A549 tumor growth in mice treated with PBS and E-M (*n* = 5 mice/group). When the tumor volume reached 100 mm^3^, PBS and E-M (12 mg/kg) were i.v. administered every other day. **(G)** A549 tumor weight in mice treated with PBS and E-M. **(H)** Representative images of immunofluorescence showing the density of F4/80^+^ macrophages in A549 tumor tissues; Blue: DAPI, green: F4/80; Scare bar = 100 μm. **(I)** Quantitation of the density of F4/80^+^ macrophages in **(H)**. **(J)** Representative images of immunofluorescence displaying the tumor angiogenesis in A549 tumor tissues via detecting CD31^+^ blood vessels; Blue: DAPI, green: CD31; Scare bar = 100 μm. **(K)** Quantified result of **(J)**. Data were representative of mean ± SD or SEM for animal experiment. *P*-value: Student’s *t*-test; ^∗∗^*P* < 0.01, ^∗∗∗^*P* < 0.001; ns, not significant.

To examine whether E-M could inhibit the recruitment of macrophages *in vivo*, we firstly implanted A549 cells into mice subcutaneously and treated mice with PBS and E-M. Six days after implantation, tumor volumes in both groups displayed no significant differences so impacts of tumor volume differences were excluded (**Figure 6A**). However, macrophage recruitment was notably inhibited in E-M-treated group, accompanied by decreased tumor angiogenesis (**Figures 6B–E**). Moreover, when the tumor volumes reached 100 mm^3^, we treated A549 tumor-bearing mice with E-M for 12 days. After the treatment, the tumor growth was significantly inhibited by E-M (**Figures 6F,G**). And we observed that the recruitment of macrophages was remarkably blocked after E-M treatment (**Figures 6H,I**). However, the density of macrophages in other organs including liver, spleen, kidney and colon from E-M treated tumor-bearing mice was not affected (Supplementary Figure 5A). Tumor angiogenesis was also significantly inhibited in E-M treatment group compared to PBS treatment group (**Figures 6J,K**). We also confirmed these results in a syngeneic tumor model. We treated B16-F10-bearing mice with E-M and got the similar results. E-M inhibited B16-F10 tumor growth and the density of intratumoral macrophages and blood vessels was significantly decreased by E-M (Supplementary Figures 5B–G). To elucidate the importance of TAMs in this process, we depleted macrophages in tumor-bearing mice with clodronate liposomes (Supplementary Figure 5H). After macrophages depletion, tumor growth and angiogenesis were both remarkably inhibited (Supplementary Figures 5I–L). In summary, E-M exhibited its inhibitory effects on tumor growth via suppression of macrophage recruitment and tumor angiogenesis.

## Discussion

ATPase activity is indispensable for the biological functions of endostatin. Nowadays, endostatin is also discovered to exhibit functions on other cell types, not only on endothelial cells. Our study, for the first time, elucidate that the engineered endostatin derivative E-M, with much higher ATPase activity, influence the recruitment and alternative activation of TAMs. Compared to the WT endostatin and other mutants with low ATPase activity, E-M exhibits much stronger inhibitory effects on macrophage recruitment. Moreover, it also inhibits the alternative activation of TAMs and M2 TAM-induced pro-angiogenic effects. These effects result in decreased intensity of intratumoral macrophages and blood vessels, which accumulatively lead to tumor regression.

In different tumor ecosystems, TAMs are the most prominent among the innate immune cells (Qian and Pollard, 2010). These macrophages are normally derived from circulating bone marrow-derived monocytes or tissue-resident macrophages (Davies et al., 2013). Chemotactic molecules such as CCL2, SCF-1, and VEGF derived from tumor cells or stromal cells mediate macrophage mobilization into tumor tissues (Lin et al., 2001; Murdoch et al., 2004; Zhang et al., 2010). Experimental and clinical studies provide evidences supporting that M2 TAMs are key regulators of tumor angiogenesis (Murdoch et al., 2008; Ruffell et al., 2012). Depletion of TAMs remarkably disrupts the blood vessel network in tumor tissues (Lin et al., 2006). Kujawski et al. (2008) once reported that activation of STAT3 in TAMs enhances the production of pro-angiogenic factors such as VEGF and bFGF. Our result was consistent with this study. A549 CM activated STAT3 and STAT6 and induced the alternative activation of macrophages, which enhanced the pro-angiogenic effects of these cells. But all these processes were blocked by E-M, indicating that E-M can inhibit tumor angiogenesis in both direct and indirect ways.

Immunosuppression is a huge challenge for anti-tumor therapy. M2-polarized TAMs are well-known to directly inhibit the immune response of CD8^+^ T cells via the production of immunosuppressive factors such as IL-10, TGF-β, and HLA-G (Wu et al., 2010). Moreover, secreted factors from tumor cells also increase the expression of programmed cell death 1 ligand (PD-L1) and cytotoxic T lymphocyte antigen 4 (CTLA-4) ligands in monocytes and macrophages (Anderson et al., 2006; Matsunaga et al., 2011; Bloch et al., 2013). Inhibitory signals from these immune checkpoints suppress the proliferation of CD8^+^ T cells and weaken the tumoricidal activity of cytotoxic T cells. Inhibition of TAMs recruitment with CSF-1R inhibitors improves the immune cell response in tumor tissues and enhances the therapeutic efficacy of chemotherapy, radiation therapy and immune checkpoint blockade therapy (Zhu et al., 2014; Shiao et al., 2015). Rolny and colleagues observed that switching macrophage polarization from M2 to M1 phenotype by histidine-rich glycoprotein (HRG) promotes vessel normalization and anti-tumor immune response, finally leading to enhanced chemotherapy and tumor growth regression (Rolny et al., 2011). Based on different experimental and clinical data, blocking TAM recruitment or targeting pro-tumor polarization of TAMs may be promising methods for cancer therapy. Endostatin gene therapy has been shown to increase cytotoxic cells including CD8^+^ T cells and NK cells in tumors from IL-2 treated mice and regulate macrophage polarization (Rocha et al., 2010; Guo et al., 2016). In our study, E-M exhibited the combined inhibitory effects on both the recruitment and alternative activation of TAMs, suggesting that it can contribute to modulating the immune microenvironment and improving the immune response. Thus targeted, cytotoxic, or immune checkpoint blockade therapy in combination with E-M may have a synergistic effect on tumor suppression.

Tumor angiogenesis is an important hallmark of cancer (Hanahan and Weinberg, 2011), and it has been widely accepted that inhibition of tumor angiogenesis as much as possible would bring the maximal survival benefit to cancer patients. However, some preclinical evidences show that targeting VEGF signaling pathway with exogenous angiogenesis inhibitors such as anti-VEGF monoclonal antibody bevacizumab and receptor tyrosine kinase inhibitors sunitinib and sorafenib elicit evasive resistance response and cause a more aggressive cancer phenotype (Ebos et al., 2009; Paez-Ribes et al., 2009). These angiogenesis inhibitors can normally prune tumor vessels efficiently and produce a hypoxic microenvironment. Hypoxia will select more malignant tumor cells which can resist these anti-angiogenic treatments (Loges et al., 2009). On the other hand, large amounts of TAMs prefer to accumulate in the poorly vascularized regions of tumor tissues (Lewis and Pollard, 2006). Besides the pro-angiogenic and immunosuppressive effects, TAMs can also facilitate tumor cell invasion and metastasis via secreting various proteolytic enzymes, cytokines and chemokines such as MMPs, cathepsins, EGF, and CCL18 (Wyckoff et al., 2004; Gocheva et al., 2010; Su et al., 2014). It may explain in part why administration of exogenous angiogenesis inhibitors promotes tumor invasiveness and metastasis. However, our findings elucidated that E-M, a derivative of the endogenous angiogenesis inhibitor endostatin, can inhibit the recruitment and alternative activation of TAMs and tumor angiogenesis simultaneously, which may well avoid these issues caused by anti-VEGF treatments. Compared to these widely used exogenous angiogenesis inhibitors, endogenous angiogenesis inhibitor E-M may have a fundamental application in the field of anti-angiogenesis treatments.

Nucleolin and integrin α5β1 are well-known co-receptor mediating the internalization of endostatin (Rehn et al., 2001; Shi et al., 2007). These proteins reportedly interact with endostatin through the heparin-binding sites of endostatin (Sasaki et al., 1999; Shi et al., 2007). As the heparin-binding sites don’t locate within the endostatin Walker A motif and E-M shows no pronounced structural changes compared to WT endostatin (Wang S. et al., 2015), we deduce that nucleolin and integrin α5β1 are the co-receptor that mediate the internalization of E-M in macrophages. And this hypothesis is validated in our study. Moreover, nucleolin and integrin α5 were both found to be up-regulated on the surface of M2 TAMs and M2 macrophages induced by IL-4 and IL-13. These increased receptors enhanced the E-M uptake and it may produce stronger inhibitory effects on M2 TAMs. Our group has reported that nucleolin is selectively expressed in angiogenic endothelia cells and lymphangiogenic vessels. And endostatin treatment had no effects on normal blood vessels and lymphatic vessels in healthy organs of tumor-bearing mice (Shi et al., 2007; Zhuo et al., 2010). Our findings observed that E-M treatment significantly decreased the density of intratumoral macrophages, but not the density of macrophages in other healthy organs such as spleen, liver, kidney and colon. As the treatment time is not long enough, we don’t know whether macrophage depletion in normal organs will also happen and whether drug toxicity will appear after a long time treatment. And this is a significant concern for studies moving forward and needs more investigation.

In summary, our study demonstrates for the first time that E-M, an engineered endostatin with higher ATPase activity, can impede the recruitment and alternative activation of TAMs. Suppression of TAM activities and tumor angiogenesis simultaneously endows E-M a stronger ability to inhibit tumor growth. Our findings illustrate that E-M is most likely to be a novel potential agent for cancer therapeutics and the ATPase activity of endostatin may provide a new approach for drug design in the future.

## Author Contributions

MX, SZ, YF, and YL conceived and designed experiments. MX, SZ, and LJ carried out the experiments. All authors contributed to the acquisition of data and analyzing the data. All authors were involved in writing the manuscript and had final approval of the submitted version.

## Conflict of Interest Statement

The authors declare that the research was conducted in the absence of any commercial or financial relationships that could be construed as a potential conflict of interest.
