# Supplementary material for: E-M, an Engineered Endostatin with High ATPase Activity, Inhibits the Recruitment and Alternative Activation of Macrophages in Non-small Cell Lung Cancer
Source: Front Pharmacol. 2017 Aug 9;8:532. doi: 10.3389/fphar.2017.00532 (PMC5552665; doi:10.3389/fphar.2017.00532)
Supplement: Supplementary file 1 [file Data_Sheet_1.DOCX]

**Supplementary Table 1**

**Oligonucleotide primer sequences used for qRT-PCR**

| Genes | Species | Oligonucleotide | Sequence (5’→3’) |  |
| --- | --- | --- | --- | --- |
| ***Vegf-A*** | **Mouse** | Upper primer  Lower primer | CTGCCGTCCGATTGAGACC  CCCCTCCTTGTACCACTGTC |  |
| ***Vegf-B*** | **Mouse** | Upper primer  Lower primer | GCCAGACAGGGTTGCCATAC  GGAGTGGGATGGATGATGTCAG |  |
| ***Pdgf-A*** | **Mouse** | Upper primer  Lower primer | TGGCTCGAAGTCAGATCCACA  TTCTCGGGCACATGGTTAATG |  |
| ***Pdgf-B*** | **Mouse** | Upper primer  Lower primer | CATCCGCTCCTTTGATGATCTT  GTGCTCGGGTCATGTTCAAGT |  |
| ***Fgf-1*** | **Mouse** | Upper primer  Lower primer | CAGCTCAGTGCGGAAAGTG  TGTCTGCGAGCCGTATAAAAG |  |
| ***Fgf-2*** | **Mouse** | Upper primer  Lower primer | GCGACCCACACGTCAAACTA  TCCCTTGATAGACACAACTCCTC |  |
| ***Angpt-1*** | **Mouse** | Upper primer  Lower primer | CTACCAACAACAACAGCATCC  CTCCCTTTAGCAAAACACCTTC |  |
| ***Angpt-2*** | **Mouse** | Upper primer  Lower primer | TTAGCACAAAGGATTCGGACAAT  TTTTGTGGGTAGTACTGTCCATTCA |  |
| ***Plgf*** | **Mouse** | Upper primer  Lower primer | AGTGGAAGTGGTGCCTTTCAA  GTGAGACACCTCATCAGGGTA |  |
| ***uPA*** | **Mouse** | Upper primer  Lower primer | GCGCCTTGGTGGTGAAAAAC  TTGTAGGACACGCATACACCT |  |
| ***Sdf-1*** | **Mouse** | Upper primer  Lower primer | GAGAGCCACATCGCCAGAG  TTTCGGGTCAATGCACACTTG |  |
| ***Tgf-β*** | **Mouse** | Upper primer  Lower primer | CCACCTGCAAGACCATCGAC  CTGGCGAGCCTTAGTTTGGAC |  |
| ***Scf*** | **Mouse** | Upper primer  Lower primer | CCTTAGGAATGACAGCAGTAGC  AGCCAATTACAAGCGAAATGAG |  |
| ***Hif-1α*** | **Mouse** | Upper primer  Lower primer | ACCTTCATCGGAAACTCCAAAG  CTGTTAGGCTGGGAAAAGTTAGG |  |
| ***Arg-1*** | **Mouse** | Upper primer  Lower primer | CTCCAAGCCAAAGTCCTTAGAG  AGGAGCTGTCATTAGGGACATC |  |
| ***Mgl-1*** | **Mouse** | Upper primer  Lower primer | TGAGAAAGGCTTTAAGAACTGGG  GACCACCTGTAGTGATGTGGG |  |
| ***18S*** | **Mouse** | Upper primer  Lower primer | CGGCTACCACATCCAAGGAA  GCTGGAATTACCGCGGCT |  |
| ***GAPDH*** | **Human &Mouse** | Upper primer  Lower primer | CAAGGTCATCCATGACAACTTTG  GTCCACCACCCTGTTGCTGTAG |  |

**Supplementary Figures**

**
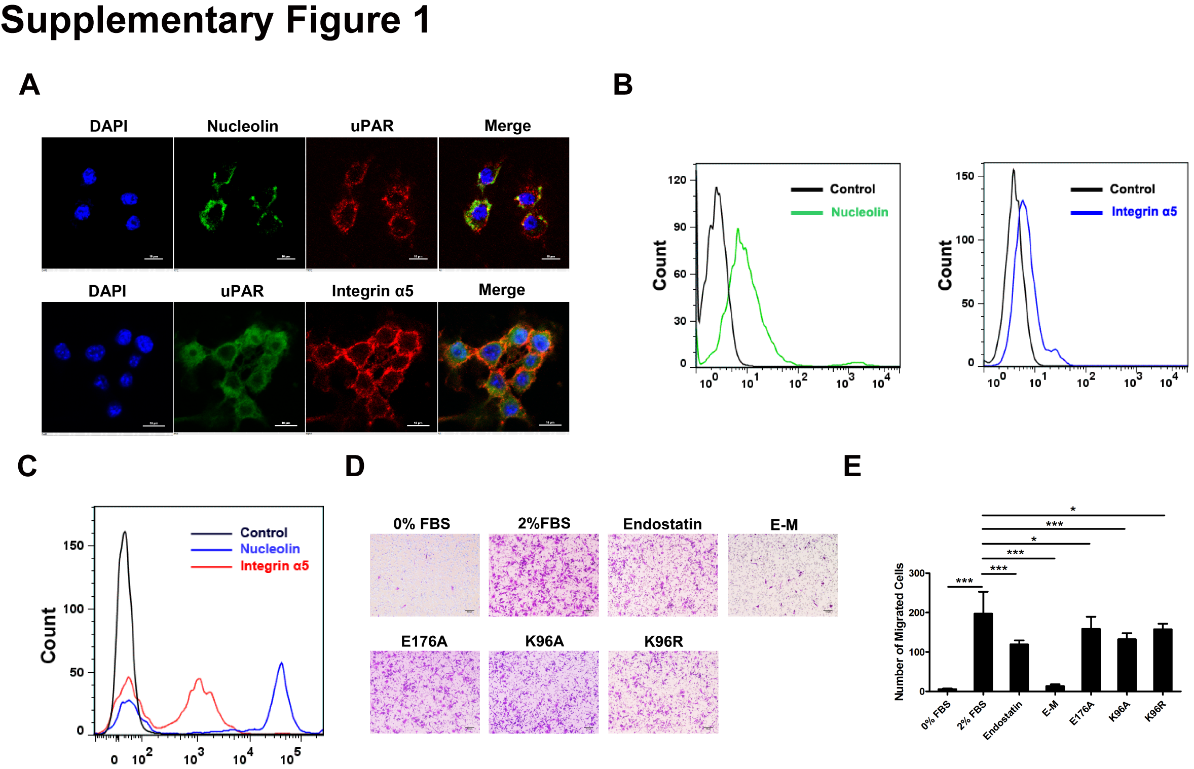
**

**Supplementary Figure 1. E-M exhibits strong inhibitory effects on Raw 264.7 cell migration.** (A) Representative images of immunofluorescence showing the expression of nucleolin, uPAR and integrin α5 on Raw 264.7 cell surface; Scare bar= 10 μm. (B) Flow cytometric analysis displaying the expression of nucleolin and integrin α5 on Raw 264.7 cell surface. (C) Flow cytometric analysis showed that nucleolin and integrin α5 were expressed on the cell surface of TAMs. TAMs were isolated from A549 xenograft tumors with CD11b-PE-Cy7 and F4/80-FITC antibodies. Then these TAMs were stained with anti-nucleolin and anti-integrin α5 antibodies. Dylight 647 conjugated-secondary antibody was used and the expression of nucleolin and integrin α5 was detected by flow cytometry. (D) Representative images of the effects of endostatin, E-M and low ATPase mutants (E176A, K96A and K96R) on Raw 264.7 cell migration determined by modified Boyden chamber assay; Scale bar=100 μm. (E) Quantified result of (D). Data were representative of mean ± SD from at least 3 independent experiments. *P* value: One-way ANOVA; ^*^*P* < 0.05, ^***^*P* < 0.001, ns: not significant.


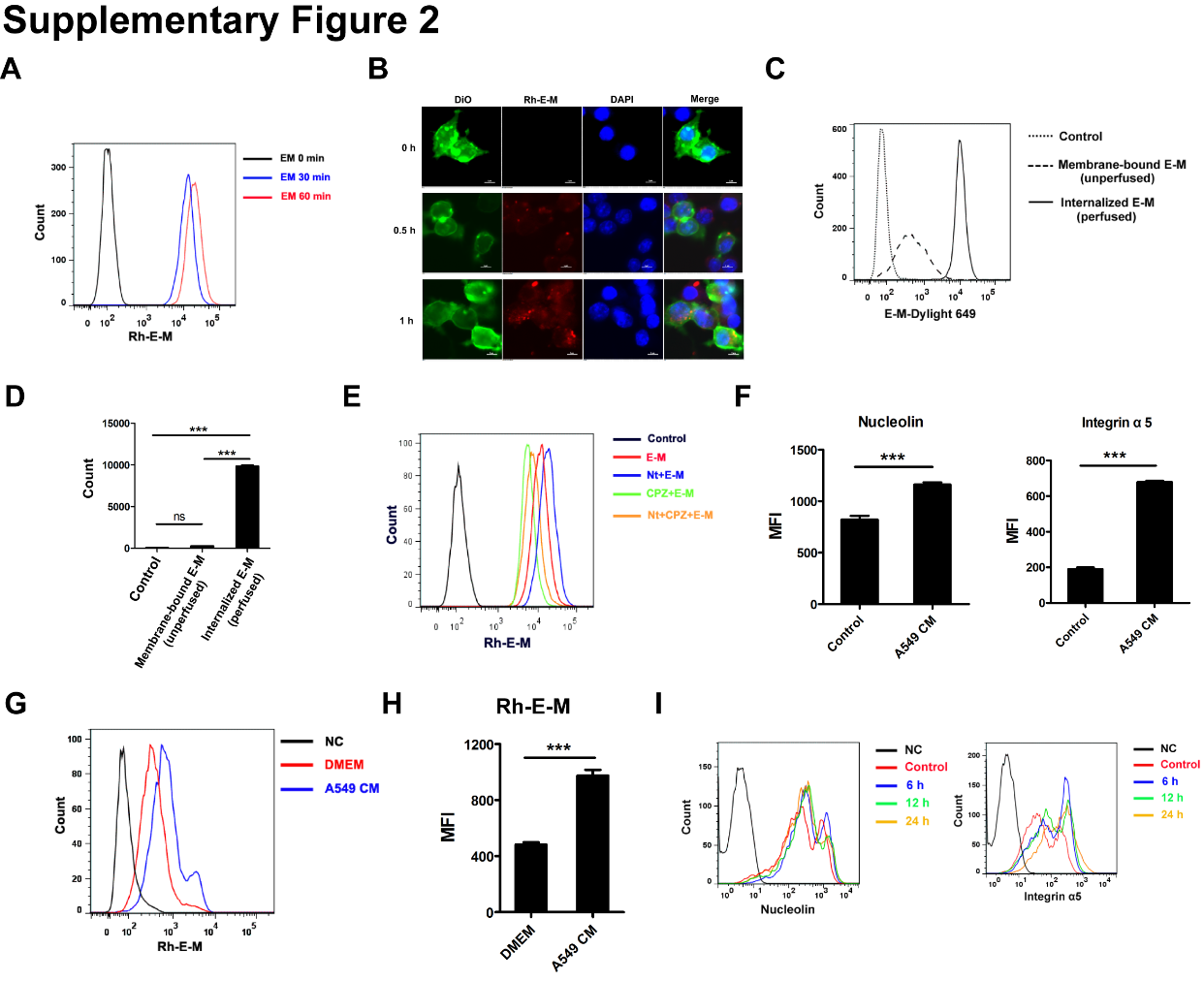


**Supplementary Figure 2. E-M can be internalized into macrophages *via* cell surface nucleolin and integrin α5β1 in caveolae/lipid raft- and clathrin-dependent pathways.** (A) Flow cytometric analysis showing the internalization of E-M into Raw 264.7 cells. (B) Representative images of immunofluorescence showing the localization of Rh-E-M in Raw 264.7 cells. Blue: DAPI, green: DiO, red: Rh-E-M; Scale bar=5 μm. (C) Flow cytometric analysis showing the localization of E-M in macrophages. To detect the membrane-bound E-M, cells were not perfused. For detecting the internalized E-M, cells were firstly washed with acid buffer and then perfused with saponin. (D) Quantified result of MFI in (C). (E) Flow cytometric analysis showing the effects of nystatin and chlorpromazine on E-M internalization in BMDMs. (F) Raw 264.7 cells were treated with A549 CM for 24 h and then the expression levels of nucleolin and integrin α5 on Raw 264.7 cell surface were detected with flow cytometry. (G) Flow cytometric result showing the internalization of Rh-E-M into Raw 264.7 cells after the treatment of A549 CM. (H) Quantified result of MFI in (G). (I) Flow cytometric analysis showing the expression levels of nucleolin and integrin α5 on BMDM surface after the treatment with IL-4 (20 ng/mL) and IL-13 (20 ng/mL) for 6, 12 and 24 h. Data were representative of mean ± SD from at least 3 independent experiments. *P* value: Student’s *t*-test for two groups and One-way ANOVA for more than two groups; ^***^*P* < 0.001, ns: not significant.


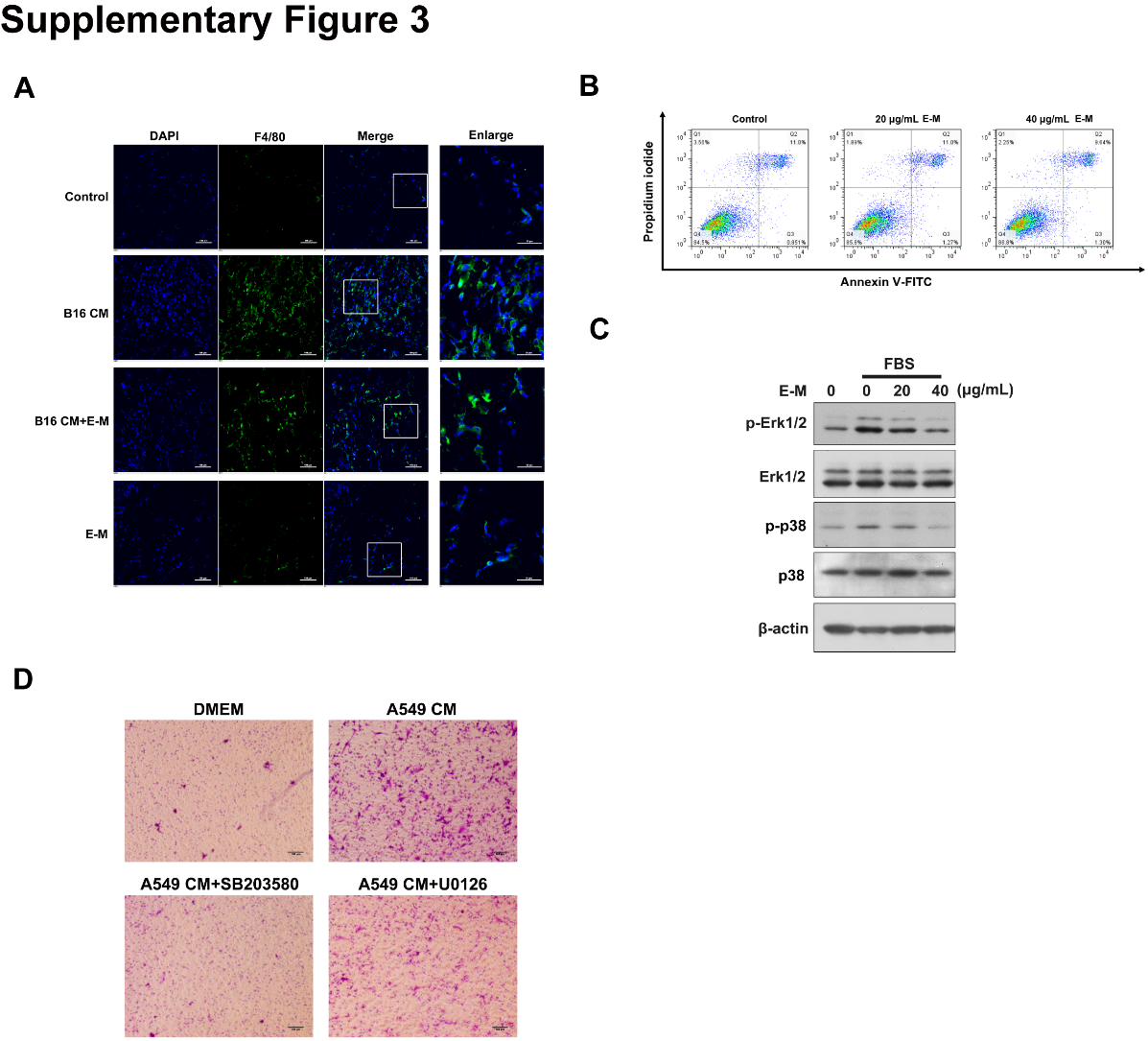


**Supplementary Figure 3. E-M suppresses the motility of macrophages through inhibition of p38 MAP kinase and Erk1/2 signaling pathways.** (A) Representative images of the density of F4/80^+^ macrophages recruited by B16-F10 CM in Matrigel plugs; Scale bar=100 μm and 50 μm for enlarged field. (B) Annexin V-FITC and PI were used to detect the effect of E-M on BMDM apoptosis. (C) Western blot showing the effect of E-M (20 μg/mL and 40 μg/mL) on the activation of Erk1/2 and p38 induced by FBS in Raw 264.7 cells. (D) Representative images of BMDM migration after the treatment of p38 inhibitor SB203580 (10 μM) and Erk1/2 inhibitor U0126 (10 μM); Scare bar= 100 μm.


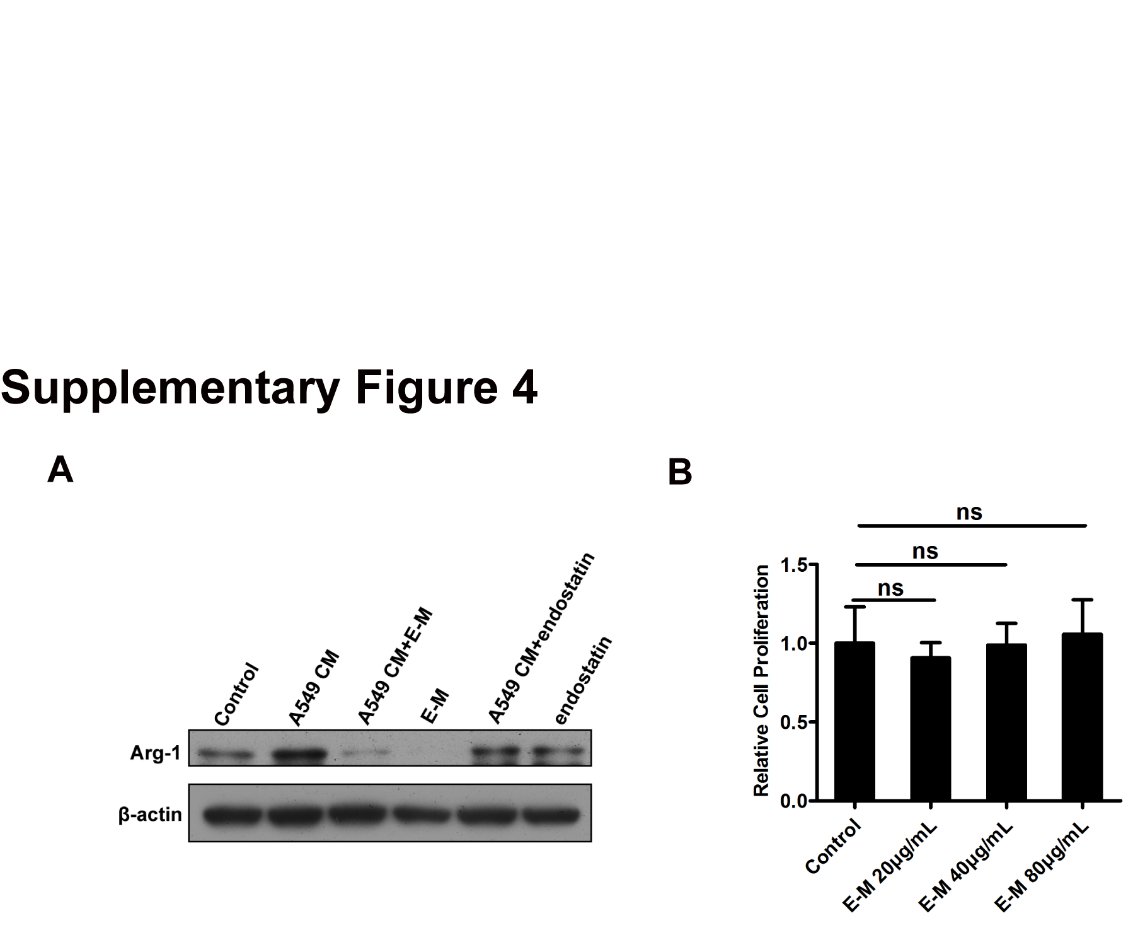


**Supplementary Figure 4. E-M inhibits the switch of macrophage polarization towards M2 phenotype.** (A) Western blotting result showing the effects of WT endostatin and E-M on inhibiting Arg-1 expression in BMDMs. (B) The effects of different concentrations of E-M (20, 40 and 80 μg/mL) on A549 cell proliferation. Data were representative of mean ± SD from at least 3 independent experiments. *P* value: One-way ANOVA; ns: not significant.


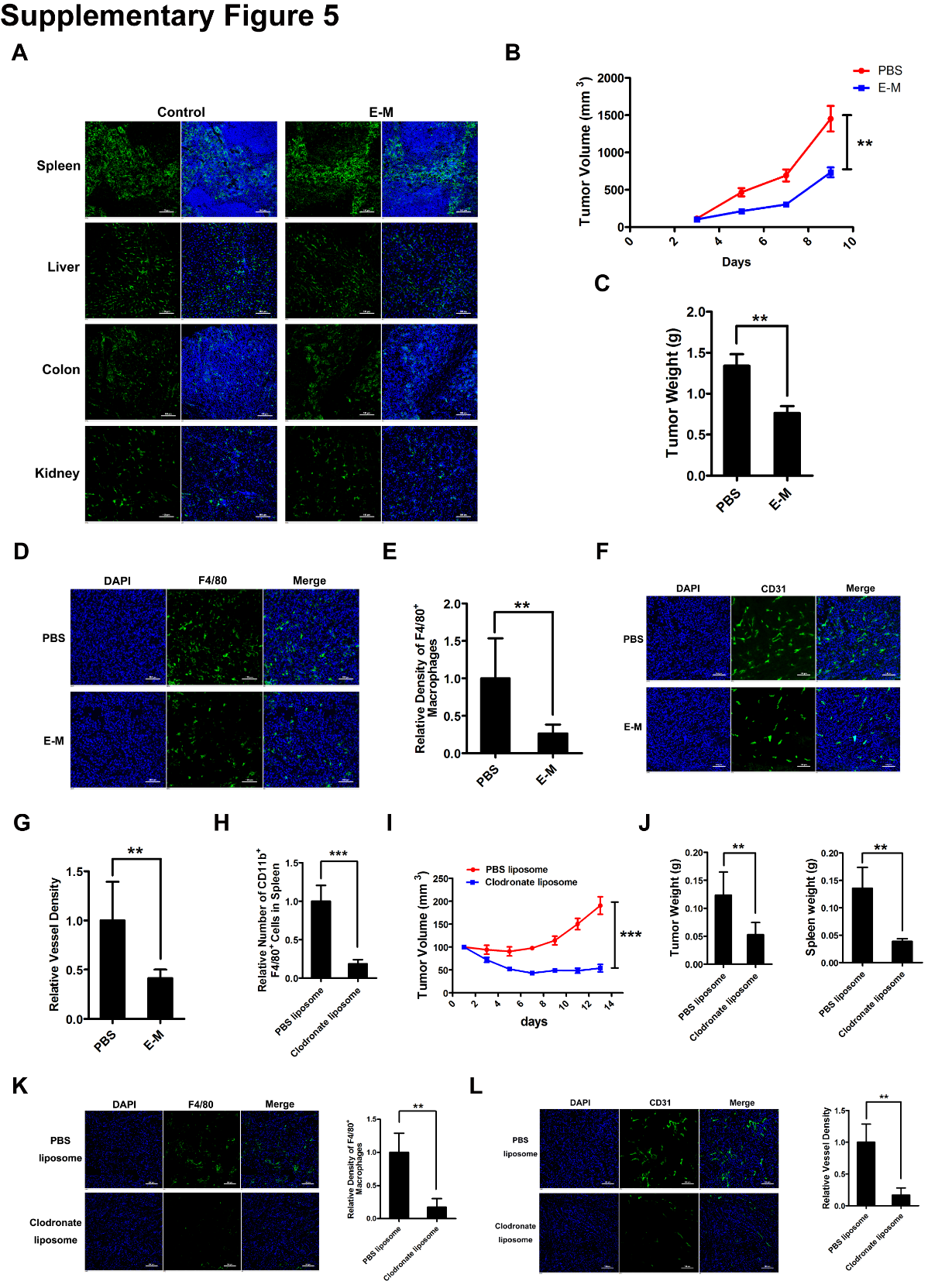


**Supplementary Figure 5. E-M inhibits the recruitment of macrophages and tumor angiogenesis *in vivo*.** (A) Representative images of immunofluorescence showing the density of F4/80^+^ macrophages in different normal organs of A549 tumor-bearing mice (n=5 mice/group); Scare bar=100 μm. (B) B16-F10 tumor growth in mice treated with PBS and E-M (n=6 mice/group). When the tumor volume reached 100 mm^3^, PBS and E-M (12 mg/kg) were i.v. administered every other day. (C) B16-F10 tumor weight in mice treated with PBS and E-M. (D) Representative images of immunofluorescence showing the density of F4/80^+^ macrophages in B16-F10 tumor tissues; Blue: DAPI, green: F4/80; Scare bar=100 μm. (E) Quantitation of the density of F4/80^+^ macrophages in (D). (F) Representative images of immunofluorescence displaying the tumor angiogenesis in B16-F10 tumor tissues *via* detecting CD31^+^ blood vessels; Blue: DAPI, green: CD31; Scare bar=100 μm. (G) Quantified result of (F). (H) Flow cytometric analysis displaying the depletion efficiency of clodronate liposomes on F4/80^+^ macrophages in the spleens of A549 tumor-bearing mice. (I) Tumor growth in A549 tumor-bearing mice after the treatment of PBS and clodronate liposomes (n=5 mice/group). (J) Tumor and spleen weights in A549 tumor-bearing mice after the treatment of PBS and clodronate liposomes. (K) Representative images of immunofluorescence and quantified result showing the density of F4/80^+^ macrophages in A549 tumor tissues after PBS or clodronate liposomes treatment; Blue: DAPI, green: F4/80; Scare bar=100 μm. (L) Representative images of immunofluorescence and quantified result showing the density of CD31^+^ blood vessels in A549 tumor tissues after PBS or clodronate liposomes treatment; Blue: DAPI, green: F4/80; Scare bar=100 μm. Data were representative of means ± SD or SEM for animal experiment. *P* value: Student’s *t*-test; ^**^*P* < 0.01, ^***^*P* < 0.001.
